# Infiltrating Monocyte Fate Switch in Retinal Degeneration: From Early Pathology to Late Homeostasis

**DOI:** 10.21203/rs.3.rs-9827841/v1

**Published:** 2026-06-18

**Authors:** Wenxin Ma, Quyan Zhang, Lijin Dong, Fusheng Tang, John Ball, Francisco M. Nadal-Nicolás, Haohua Qian, Rafael Villasmil, Pradeep Dagur, Johnny Tam, Wai T Wong, Wei Li

**Affiliations:** National Eye Institute; National Eye Institute; National Eye Institute; National Eye Institute; National Eye Institute; National Eye Institute; National Eye Institute; National Eye Institute; National Heart Lung and Blood Institute; National Eye Institute; National Eye Institute; National Eye Institute

**Keywords:** Microglia, macrophage, MDM, MiDM, Müller cell, C3, CFH, CCR2-CreER

## Abstract

The contribution of infiltrating monocyte-derived macrophages (MDMs) to neurodegeneration remains poorly understood. Using a CCR2-CreER–based lineage-tracing and ablation strategy in mouse models of retinal degeneration, we precisely tracked infiltrating monocytes and defined their functional evolution. We found that monocytes entering the degenerating retina rapidly downregulate CCR2 and progressively acquire microglial markers (e.g., TMEM119, P2RY12), adopt a ramified morphology, and acquire a transcriptional signature highly analogous to that of resident microglia-derived macrophages (MiDMs). While a subset of monocytes is cleared by activated microglia during the acute phase, others persist and integrate into the retinal macrophage niche, restoring myeloid homeostasis in later stages. Functionally, early monocyte infiltration is pathogenic; it amplifies neuroinflammation by inducing Müller cells to produce complement C3 and shifts microglial complement regulation toward the alternative pathway. Critically, selective ablation of monocytes during this acute phase attenuated C3 deposition, suppressed microglial activation, and preserved photoreceptors. Together, these findings reveal a temporally bifurcated role for infiltrating monocytes—as early drivers of complement-mediated pathology and later contributors to immune homeostasis—and identify monocyte infiltration as a stage-specific therapeutic target in neurodegenerative disease.

## Introduction

Neuroinflammation is a critical component of nearly all neurodegenerative diseases, yet targeting it therapeutically has proven challenging^1–6^. Broad immunosuppression often fails in clinical trials^7,8^, suggesting that a more precise understanding of the specific cellular players and their temporal roles is required to develop effective treatments^3,9,10^. Infiltrating peripheral monocytes are central to this challenge; they are recruited to sites of central nervous system (CNS) injury, including the retina, in diseases such as age-related macular degeneration^11–13^, but their contribution remains intensely debated^14–16^. Whether these cells are pathogenic drivers that should be blocked^17–20^ or reparative agents that should be promoted^21,22^ remains a fundamental unanswered question, creating a major roadblock for the design of targeted immunomodulatory therapies^3,23,24^.

This controversy stems from a critical technical limitation: monocytes rapidly downregulate canonical surface markers like CCR2 upon entering tissue, making it nearly impossible to track their long-term fate and function with conventional methods^25–29^. This has obscured their true role, leaving their potential as a therapeutic target unrealized. The retina, with its well-defined cellular architecture and clinical accessibility, offers an ideal system for resolving this question and modeling the dynamics of neuroinflammation in the CNS^30–32^.

Currently, two peripheral monogranular cell-specific tracing mouse lines have been used^29,33–35^. Here, we also generated and used an inducible CCR2-CreER fate-mapping system to definitively track and functionally interrogate infiltrating monocytes in mouse models of retinal degeneration. We provide conclusive evidence that these cells have a dual, stage-dependent role. Initially, they are profoundly pathogenic, orchestrating a neurotoxic inflammatory cascade by activating the alternative complement pathway. Critically, we show that blocking their entry during this acute phase is a robustly neuroprotective strategy that preserves both retinal structure and function. Subsequently, the persisting monocytes transition to a reparative, homeostatic state. By defining the precise pathogenic contribution of acute monocyte infiltration, our findings resolve a key controversy in neuro-immunology and, most importantly, identify a specific and actionable therapeutic window for treating a host of complement-driven neurodegenerative diseases.

## Results

### Infiltrating monocytes transiently express CCR2 and Ly6C but can be indelibly tracked using a CCR2-CreER fate-mapping system

**Figure 1:**
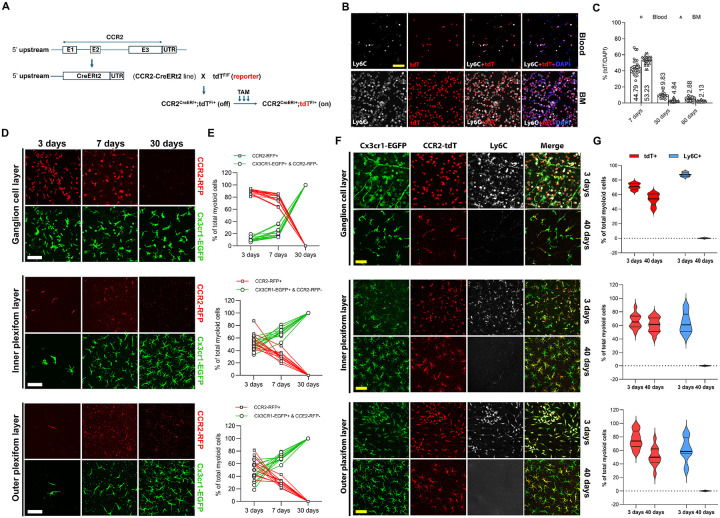
A CCR2-CreER fate-mapping system enables long-term tracking of monocyte-derived macrophages (MDMs). (A) Schematic of the CCR2-CreER knock-in strategy, where the CreERT2 coding sequence replaces the endogenous CCR2 sequence. CCR2^CreER/CreER^ mice were crossed with a tdTomato (tdT) reporter line to generate CCR2^CreER/+^;tdT^F/+^ mice, enabling tamoxifen (TAM)-inducible labeling of CCR2-expressing cells. (B) Representative images showing efficient tdT expression (red) in Ly6C+ monocytes (white) within the blood and bone marrow (BM) one week post-tamoxifen induction. Nuclei are counterstained with DAPI (blue). Scale bar: 60 μm. (C) Quantification of tdT+ cells in the blood and bone marrow at 7, 30, and 60 days post-induction, demonstrating the rapid turnover of the labeled peripheral monocyte pool. (D) and (E) Conventional CCR2-RFP reporter mice fail to track monocytes long-term. In CCR2^RFP/+^;CX3CR1^EGFP/+^ mice subjected to NaIO_3_-induced retinal injury, RFP-expressing cells (red) infiltrate the retina at 3 and 7 days but are undetectable by day 30, demonstrating the transient nature of CCR2 expression. Scale bar: 60 μm. (F) and (G) The CCR2-CreER system provides indelible long-term tracking. In CCR2^CreER/+^;tdT^F/+^;CX3CR1^EGFP/+^ mice, fate-mapped tdT+ MDMs (red) persist in the retina from the acute phase (3 days) to the chronic phase (40 days) post-injury. These persistent MDMs downregulate the transient monocyte marker Ly6C (white), which is only expressed at the 3-day time point. Scale bar: 40 μm.

To definitively track the fate of peripheral monocytes infiltrating the degenerating retina, we generated a CCR2-CreER mouse line by replacing the CCR2 coding sequence with a sequence coding for CreERT2, a tamoxifen-inducible Cre recombinase protein^36^ (Fig. 1A). These mice were then crossed to tdtomato (tdT) reporter strain^37^ to generate heterozygous CCR2^CreER/+^;tdT^F/+^ mice, which were used for the experiments. We confirmed the absence of Cre recombinase leakage (Fig. S1, A-B). Upon induction with tamoxifen administration, tdT expression was detected in 44.79% and 53.23% of granulocytes in the blood and bone marrow, respectively, at 1-week post-induction (Fig. 1B–C). As the turnover of monocytic cells in the blood and bone marrow is important for identifying resident, long-lived microglia in the CX3CR1^CreER/+^;tdT^F/+^ model^25,26^, we assessed the extent of tdT+ cell turnover in blood and bone marrow. We found that the proportions of tdT+ cells decreased progressively with time to 9.83% and 4.84% in the blood and bone marrow, respectively, at 1 month, and then to 2.88% and 2.13% at 2 months post-induction (Fig. 1C). In the healthy adult mouse retina, tdT+ cells were absent (Fig. S1C). In contrast, numerous tdT+ cells were observed in retinal degeneration, including the sodium iodate (NaIO_3_)-induced retinal pigment epithelial (RPE) cell injury model, the rd10 photoreceptor degeneration model, and the light-induced photoreceptor damage model (LD) (Fig. S1D). These results demonstrate that the CCR2-CreERt2 mouse line is suitable for tracking peripheral monocyte infiltration from the bone marrow and blood into the injured retina during retinal degeneration.

Previous studies have shown that monocyte-derived macrophages (MDMs) normally resident in the central nervous system (CNS) are joined by CCR2+, LY6C+ monocytes recruited from the systemic circulation under inflammatory conditions; however, these recruited cells were initially thought to be cleared from the retina as immunopositivity for CCR2 and Ly6C cell markers declined with time^20,26,38,39^. To further understand the fate of MDMs in the context of retinal degeneration, we used the NaIO_3_-induced RPE injury model, a mimic of dry age-related macular degeneration (AMD). We first used a conventional CX3CR1^EGFP/+^;CCR2^RFP/+ 40^ double-transgenic mouse to trace infiltrating monocytes (which are marked by both RFP and low EGFP). Consistent with previous reports, RFP+ monocytes infiltrated the retina on day 3 but were undetectable by day 30, suggesting their clearance (Fig. 1D–E). However, this observation cannot distinguish between cell removal and the mere downregulation of the CCR2 reporter. We resolved this ambiguity using our CCR2-CreER fate-mapping system, in which tdT expression is permanent after induction, regardless of ongoing CCR2 expression. In this model, tdT+ MDMs were present not only on day 3 but also for at least 40 days post-injury (Fig. 1F–G). We further observed that these persistent tdT+ MDMs were positive for the monocyte marker Ly6C at day 3 but became Ly6C-negative by day 40 (Fig. 1F–G). Together, these findings demonstrate that infiltrating monocytes downregulate canonical markers such as CCR2 and Ly6C shortly after tissue entry, and that previous conclusions about their disappearance were confounded by reliance on transient reporters^25,26^. Our CCR2-CreER system provides an indelible tag that definitively proves these cells persist long-term within the retinal tissue.

#### Persistent MDMs acquire a microglial identity and integrate into the retinal myeloid niche

**Figure 2:**
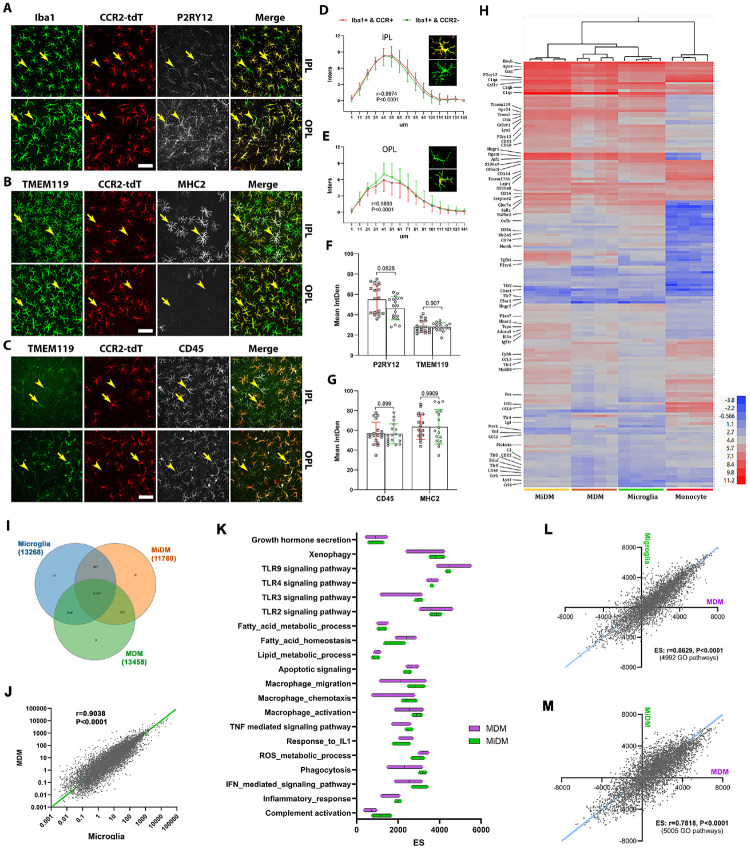
Persistent MDMs acquire a microglial identity and integrate into the retinal myeloid niche at the steady state post-injury. (A) Representative retinal flat-mount images at 40 days post-NaIO_3_ injury. Fate-mapped CCR2-tdT+ MDMs (arrows) and resident CCR2-tdT- MiDMs (arrowheads) both express the myeloid marker Iba1 (green) and the microglial marker P2RY12 (white), forming a contiguous mosaic in the inner (IPL) and outer (OPL) plexiform layers. Scale bar: 60 μm. (B) & (C) Both MDMs (arrows) and MiDMs (arrowheads) co-express the microglial signature marker TMEM119 (white) as well as the activation markers MHC Class II (MHC2, green) and CD45 (cyan) at 40 days post-injury. Scale bar: 60 μm. (D) & (E) Sholl analysis (Image J) reveals no significant morphological differences between MDMs and MiDMs in either the IPL (D) or OPL (E), indicating a shared ramified structure. (F) & (G) Quantification of mean fluorescence intensity confirms no significant difference in the expression levels of P2RY12 and TMEM119 (F) or CD45 and MHC2 (G) between MDMs and MiDMs. (H). Hierarchical clustering of 189 microglia-enriched genes (Supplement file 1) shows that the transcriptomic profile of MDMs is highly similar to that of MiDMs and closely resembles that of resting microglia. (JMP 16 was used). (I). Venn diagram illustrating the extensive overlap in gene expression between resting microglia and MDMs, with the fewest differentially expressed genes (394) found between MDMs and resting microglia. InteractiVenn was used (https://www.interactivenn.net/)^63^. (J). Correlation analysis reveals a strong positive correlation (r=0.9038, P<0.0001) between the gene expression profiles of MDMs and resting microglia (Supplement file 2). GrahPad Prism 10 was used. (K) Comparison of microglia major functional enrichment scores between MiDMs and MDMs. Gene Ontology (GO) biological processes were quantified using single-sample Gene Sets Enrichment Analysis (ssGSEA)^64^. (L, M) Correlation analysis of ssGSEA-derived pathway enrichment scores (Supplement file 3). The global functional profiles of MDMs are plotted against resting microglia (L) and MiDMs (M) to demonstrate phenotypic convergence.

Having established that infiltrating monocytes persist over the long term, we next investigated their fate during the chronic, stable phase of injury. At 40 days post-injury, a time point corresponding to the reestablishment of myeloid homeostasis^26,29^, fate-mapped CCR2-tdT+ MDMs were found organized in a contiguous mosaic alongside CCR2-tdT unlabeled, resident microglia-derived macrophages (MiDMs)^41^ in the retinal plexiform layers (Fig. 2A). Morphologically, the two populations were indistinguishable; both displayed a highly ramified structure with no significant difference in cellular morphology as revealed by Sholl analysis (Fig. S2A, 2D-E).

Immunohistochemical analysis of myeloid marker expression revealed that persistent MDMs acquired expression of canonical microglial signature markers, including P2RY12 and TMEM119, at levels comparable to adjacent MiDMs (Fig. 2A–B, 2F). This acquisition of a microglial phenotype by tdT+ MDMS was observed across multiple injury models, including light-induced damage and inherited degeneration (Fig. S2B, S2D), indicating a generalized response. Furthermore, both tdT+ MDMs and tdT- MiDMs expressed similar levels of the activation markers MHC Class II and CD45, suggesting that they occupy a similar functional state (Fig. 2B–C, 2G; S2C-D).

To confirm this convergence at the transcriptomic level, we performed bulk RNA-seq on Fluorescence-Activated Cell Sorting (FACS)-sorted tdT+ MDMs and CD11b + tdT- MiDMs isolated from the retina 40 days post-NaIO_3_ injury. Unsupervised clustering based on a panel of microglia-enriched genes (Supplement file 1) revealed that MDMs were highly analogous to both MiDMs and resting microglia (Fig. 2H). Strikingly, the gene expression profile of MDMs was more closely correlated with that of resting microglia than that of MiDMs (Fig. 2H–J, Supplement file 2). Gene Ontology (GO) with single-sample gene set enrichment analysis (ssGSEA) confirmed this functional similarity, showing no significant differences in pathways related to inflammation, phagocytosis, or complement, the key functions of microglia, between MDMs and MiDMs at this chronic stage (Fig. 2K). The global functional profiles analysis using the enrichment scores of MDMs to resting microglia and MiDM (Supplement file 3), the results indicated that MDM are closer to resting microglia (Fig. 2L, r = 0.8629, P < 0.0001) than MiDMs (Fig. 2M, r = 0.7818, P < 0.0001) to demonstrate phenotypic convergence. Collectively, these data demonstrate that infiltrating monocytes undergo a complete phenotypic and transcriptomic reprogramming, differentiating to resemble resident microglia as they integrate into and restore the retinal myeloid niche.

#### Acute monocyte infiltration drives a pathogenic microglial response characterized by clustering and phagocytosis

The MDMs persisted in the retina and integrated into myeloid homeostasis in the later, stable stage after injury. Here, we assessed the relationship between MiDMs and MDMs at the acute phase of the retinal injury. To distinguish the later-stable-stage MiDM and MDM, we refer to them as aMiDM and aMDM (acute phase). We used the rd10 model of inherited retinal degeneration^42^. The rd10 mice have a spontaneous missense mutation in PDE6B and have been used as a model of Retinitis pigmentosa (RP). We generated the rd10;CCR2^CreER/+^;tdT^F/+^ and rd10:CCR2^CreER/+^;tdT^F/+^;DTA^F/+^ mouse lines to track and manipulate MDMs. After tamoxifen administration, CCR2 + cells will be labeled with tdTomato in both lines, but depletion of CCR2 + cells will occur only in the rd10:CCR2^CreER/+^;tdT^F/+^;DTA^F/+^ mouse line, as referred to as “depletion” thereafter. The group of rd10:CCR2^CreER/+^;tdT^F/+^ mouse line referred to as “no depletion” thereafter. First, we examined the efficiency of DTA-mediated depletion. The results showed that DTA-mediated depletion of CCR2 + cells was highly efficient, leading to a near-complete absence of monocyte infiltration into the retina (Fig. S3A-G).

**Figure 3:**
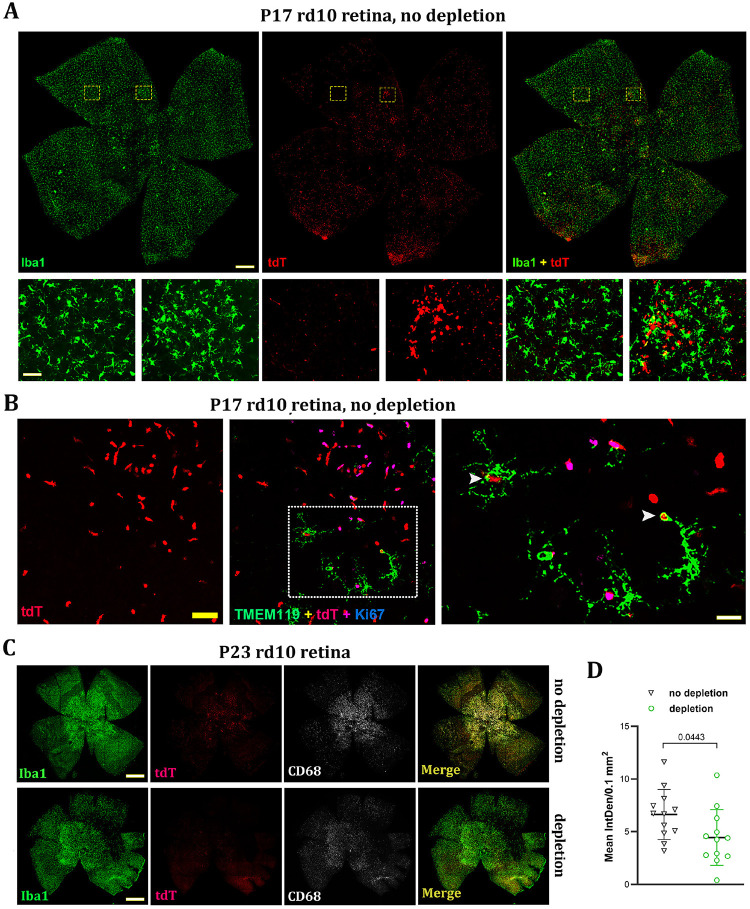
Acute monocyte infiltration drives resident microglial clustering and enhances phagocytic activity. (A) Representative retinal flat-mount images from P17 rd10 mice, illustrating that the presence of tdT+ acute monocyte-derived macrophages (aMDMs, red) induces clustering of Iba1+ acute microglia-derived macrophages (aMiDMs, green). The upper panel shows a broad overview, while the lower panels provide magnified views of areas with and without aMDMs, highlighting how aMDMs disrupt even microglial distribution. Scale bars: 1.2 mm (top), 160 μm (bottom). (B) Magnified images demonstrating active interactions between aMiDMs and aMDMs. Infiltrating CCR2+ cells (tdT+, red) are shown co-localizing with TMEM119+ aMiDMs (green), with an arrowhead pointing to an aMiDM actively phagocytosing a CCR2+ cell. Scale bars: 124 μm (left), 53 μm (right). (C) Whole retinal flat-mount images of P23 rd10 mice, comparing CD68 immunoreactivity (white), a marker for phagocytic activity, in retinas with and without monocyte depletion. Scale bar: 1.2 mm. (D) Quantification of mean CD68 intensity, demonstrating a significant decrease in microglial phagocytic activity in rd10 retinas following monocyte depletion.

In rd10 mice, rods begin to degenerate around P16, with maximum cell death occurring between P21 and P25. Therefore, tamoxifen was administered at P14-P16 before degeneration initiation. In the non-depletion group of rd10 retinas on postnatal day 17 (P17), during the early stage of degeneration, infiltration by aMDMs triggered a dramatic change in the behavior of resident aMiDMs. The normally organized microglial mosaic was disrupted as aMiDMs migrated and formed dense clusters around the newly arrived aMDMs (Fig. 3A). This clustering was entirely dependent on monocyte infiltration, as it was absent in monocyte-depleted mice (Fig. S4A). High-resolution imaging revealed an antagonistic relationship within these clusters, with aMiDMs actively phagocytosing the infiltrating aMDMs (Fig. 3B, S4B). The presence of aMDMs also induced a broader microglial activation, evidenced by a significant increase in CD68 expression throughout the retina (Fig. 3C–D). This aggressive interaction correlated with increased retinal damage; the presence of infiltrating monocytes led to a significant loss of cone photoreceptors, as quantified by peanut agglutinin (PNA) staining (Fig. S4C-D). Together, these findings demonstrate that in the acute phase of degeneration, infiltrating monocytes are not cooperative but instead trigger a pathological microglial activation state that exacerbates neurodegeneration.

### Infiltrating monocytes reprogram resident microglia to activate the alternative complement pathway and increase the detrimental conditions in the acute phase of retinal degeneration

**Figure 4:**
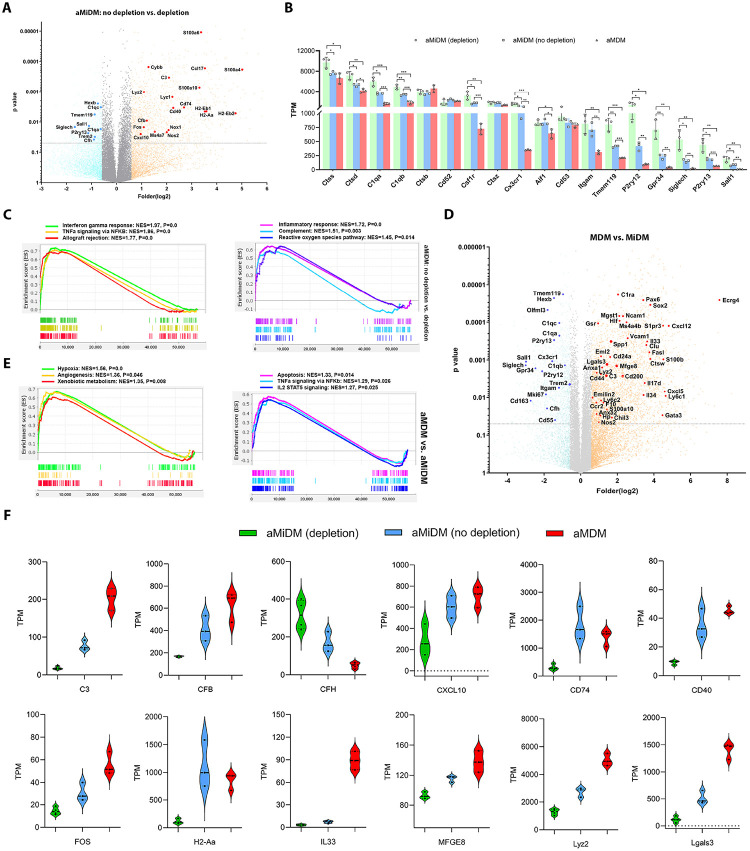
Bulk RNA sequencing reveals that acute monocyte infiltration drives resident microglial activation and distinct functional profiles between aMiDMs and aMDMs. (A) The volcano plot showed differential gene expression in aMiDM between no depletion vs. depletion in P23 rd10 mice. (B) The diagram displays the expression levels of key microglial signature genes in aMiDMs from the depletion group, aMiDMs from the non-depletion group, and aMDMs from the no depletion group in P23 rd10 mice retinas. (C) Gene Set Enrichment Analysis (GSEA) comparing gene expression pathways in aMiDMs under conditions of no depletion versus the depletion group of retinas (Supplement file 4). (D) The volcano plot revealed distinct gene expression patterns between aMDM and aMiDM within the same retina in P23 rd10 mice. (E) GSEA comparing gene expression pathways between aMDMs and aMiDMs in P23 rd10 retinas (Supplement file 5). (F) Violin plots illustrating the expression distribution of representative genes identified as significant in the GSEA results from panels (C) and (E).

To define the molecular mechanisms underlying the pathogenic acute-phase interaction, we performed bulk RNA-seq on FACS-sorted acute microglia-derived macrophages (aMiDMs; CD11b + tdT-) and acute monocyte-derived macrophages (aMDMs; tdT+) from P23 rd10 retinas. By comparing aMiDMs from the no depletion group versus the depletion group, we found that the presence of aMDMs fundamentally altered the microglial transcriptome. Specifically, aMiDMs in non-depleted retinas lost their homeostatic signature, downregulating key microglial identity genes like *Tmem119, P2ry13, Hexb, C1qa, Csf1r, Trem2* (Fig. 4A–B). Concurrently, these aMiDMs exhibited a potent pro-inflammatory profile, including inflammation and interferon gamma response genes such as *Cd74, Cxcl10, Cd40, Ccl17, Nos2*, and *H2-Aa*; hypoxia-related genes *Fos, S100a4*; and phagocytosis genes *Lyz1, Lyz2*, and *Cybb* (Fig. 4A, 4F; Fig. S5A). Gene Set Enrichment Analysis (GSEA)^43^ revealed a strong upregulation of pathways related to interferon signaling, TNF-α signaling, and reactive oxygen species (ROS) production (Fig. 4C; Supplement file 4). We confirmed this inflammatory reprogramming at the protein level, demonstrating significantly higher expression of CD74 and MHC Class II in aMiDMs only when aMDMs were present (Fig. S5B-E). Most critically, the presence of aMDMs induced a profound shift in the complement system of aMiDMs to activation (Fig. 4C; Supplement file 5), characterized by a significant upregulation of complement component *C3* and the alternative pathway activator Factor B (*Cfb*), alongside a downregulation of the key alternative pathway inhibitor, Factor H (*Cfh*) (Fig. 4A, 4F). This transcriptional shift strongly indicates that infiltrating monocytes instruct resident microglia to activate the alternative complement cascade (Fig. 4C).

Direct comparison of the aMDMs and aMiDMs transcriptomes from the same non-depleted retinas revealed distinct functional roles. While aMDMs expressed some microglial genes (Fig. 2B), they were primarily characterized by high expression of classic monocyte/macrophage markers (*Ccr2, Ly6c1, Spp1*) and genes associated with cell migration and proliferation (Fig. 4D; Fig. S5A). GSEA of aMDMs showed enrichment for pathways like xenobiotic metabolism, potentially reflecting their “foreign” status that triggers their phagocytosis by aMiDMs (Fig. 4E). Taken together, these data demonstrate that in the acute phase of degeneration, infiltrating aMDMs are not passive bystanders but act as potent signaling hubs that reprogram resident microglia, suppressing their homeostatic function and driving a pathogenic, complement-mediated inflammatory state.

#### Ablation of infiltrating monocytes is profoundly neuroprotective in models of retinal injury

**Figure 5:**
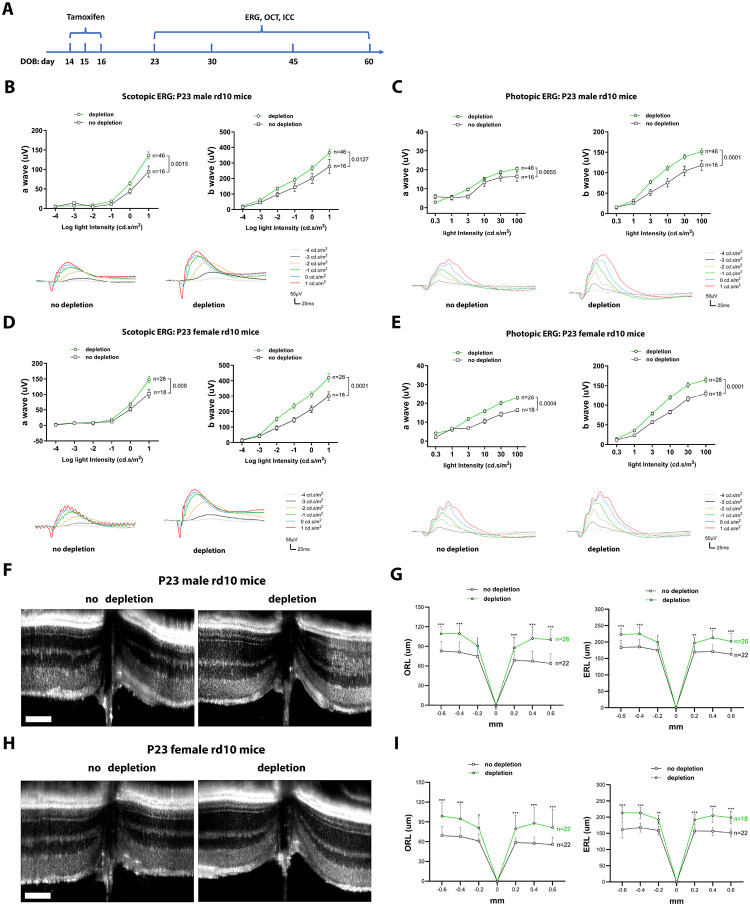
Ablation of peripheral monocytes protects retinal structure and function in models of retinal degeneration. (A) Experimental timeline illustrating tamoxifen administration, assessment time points for electroretinography (ERG), optical coherence tomography (OCT), and immunocytochemistry (ICC). (B) & (C) Electroretinography (ERG) results from P23 male rd10 mice. The top panels show plots of a- and b-wave amplitudes across various light intensities for scotopic (rod-mediated) (B) and photopic (cone-mediated) (C) conditions. The bottom panels display representative mean ERG waveforms for each light intensity. Monocyte depletion significantly improved both rod and cone function. (D) & (E) ERG results from P23 female rd10 mice. The top panels show plots of a- and b-wave amplitudes across various light intensities for scotopic (rod-mediated) (D) and photopic (cone-mediated) (E) conditions. The bottom panels display representative mean ERG waveforms for each light intensity. Monocyte depletion significantly improved both rod and cone function. (F) Representative spectral domain optical coherence tomography (SD-OCT) images of P23 male rd10 retinas from superior to inferior, comparing non-depleted (left) and monocyte-depleted (right) conditions. Scale bar: 0.1 mm. (G) Quantify the thickness of the outer retinal layer (ORL) and the entire retinal layer (ERL), demonstrating significant structural preservation with monocyte depletion in p23 male mouse retinas. (H) Representative SD-OCT images of P23 female rd10 retinas from superior to inferior, comparing non-depleted (left) and monocyte-depleted (right) conditions. Scale bar: 0.1 mm. (I) Quantify the thickness of the ORL and the ERL, demonstrating significant structural preservation with monocyte depletion in p23 female mouse retinas. All ERG data were analyzed with 2-way ANOVA, and the retinal thickness data were analyzed with an unpaired two-tailed Student’s t-test. * p<0.05, **P<0.01, *** P<0.001.

Given that acute monocyte infiltration drives a pathogenic inflammatory state, we hypothesized that blocking their entry would be neuroprotective. To test this, we ablated CCR2 + monocytes in rd10 mice just before the start of photoreceptor degeneration (tamoxifen administration from P14-P16) and assessed retinal function and structure at different time points after (Fig. 5A). At P23, monocyte-depleted mice exhibited a significant preservation of retinal function. Electroretinography (ERG) revealed significantly higher a-wave and b-wave amplitudes under both scotopic and photopic conditions in depleted mice of both sexes, indicating robust protection of both rod and cone pathways, as well as inner retinal function (Fig. 5B–E). This functional preservation was sustained at P30 (Fig. S6A-B). This functional protection was directly correlated with the preservation of retinal structure. To examine structure, we measured retinal thickness using spectral domain optical coherence tomography (SD-OCT)^42,44^. To measure the thickness of the outer retina layer (ORL) and the entire retinal layer (ERL) more easily and precisely, the superior and inferior retina were measured. The ORL was measured from the RPE apical to the OPL, including the OPL; the ERL was measured from the RPE apical to the ganglion cell layer/retinal nerve fiber (GL/RNF). SD-OCT at P23 showed that the outer retinal layer (ORL) and entire retinal layer (ERL) were significantly thicker in monocyte-depleted mice compared to non-depleted controls (Fig. 5F–I). This structural preservation also persisted to P30 (Fig. S6C-D), confirming that preventing monocyte infiltration provides a durable neuroprotective effect.

To determine whether this protective effect was specific to the inherited degeneration model, we repeated the experiment using the NaIO_3_-induced RPE injury model. Consistent with our findings in rd10 mice, monocyte depletion resulted in significant preservation of retinal thickness at both 3 and 8 days post-injury (Fig. S6E-F). Taken together, these results demonstrate that blocking the infiltration of peripheral monocytes exerts a general protective effect against retinal degeneration.

#### Monocyte ablation prevents pathogenic complement activation by restoring Müller cell and microglial homeostasis

**Figure 6:**
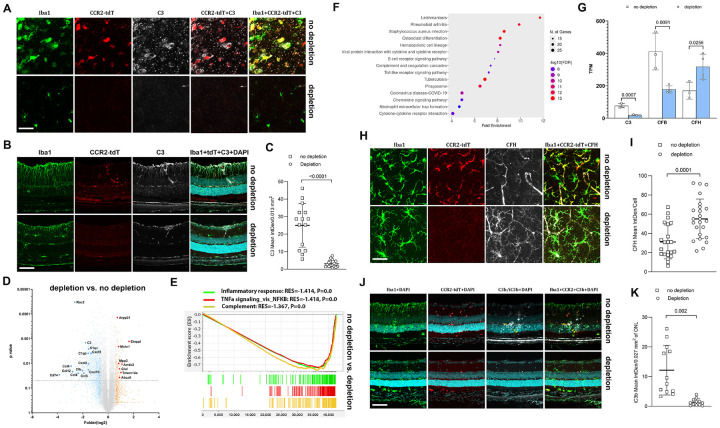
Monocyte ablation attenuates Müller cell C3 production and suppresses alternative complement activation. (A) Representative retinal flat-mount images of the outer nuclear layer (ONL) from P19 rd10 mice, stained for Iba1 (green) and C3 (white). Fate-mapped aMDMs are labeled with tdTomato (red). In non-depleted retinas, C3 is expressed in aMiDMs, aMDMs, and notably, Müller cells. C3 expression is significantly reduced following CCR2+ cell depletion. Scale bar: 30 μm. (B) Retinal cross-sections from P23 rd10 mice, stained for Iba1 (green) and C3 (white), confirming that C3 expression in Müller cells and deposition on photoreceptors are markedly decreased in monocyte-depleted retinas compared to non-depleted controls. Scale bar: 60 μm. (C) Quantification of mean C3 intensity in the ONL, corresponding to data shown in (A), confirming a significant reduction in C3 expression after monocyte depletion. (D) Volcano plot showing differential gene expression (DGE) in CD11b- and CCR2-tdT-negative retinal cells (primarily Müller cells and astrocytes) between monocyte-depleted and non-depleted groups. (E) Gene Set Enrichment Analysis (GSEA) of the CD11b- and CCR2-tdT-negative retinal cell population, comparing non-depleted condition versus monocyte-deleted condition. (F) Gene Ontology (GO) analysis of significantly increased genes in the CD11b- and CCR2-tdT-negative retinal cells in the non-depletion group compared to the depletion group. ShinyGO 0.82 was used (https://bioinformatics.sdstate.edu/go82/). (G) Expression levels of complement C3 and its regulators, CFB and CFH, in retinal CD11b+ but CCR2-tdT- aMiDMs from P23 rd10 mice. (H) Retinal flat-mount images stained for Iba1 (green) and CFH (white) from P23 rd10 non-depleted and depleted mice. CFH expression in aMiDMs is significantly decreased in the presence of infiltrating monocytes. Scale bar: 30 μm. (I) Quantification of the mean CFH intensity per aMiDM, showing reduced CFH in non-depleted retinas. (J) C3b/iC3b staining (white) demonstrates significantly increased C3b/iC3b deposition on aMiDMs (arrow) and photoreceptors (arrowheads) in non-depleted rd10 retinas compared to monocyte-depleted retinas. Scale bar: 60 μm. (K) Quantification of the mean C3b/iC3b intensity in the ONL, confirming significantly reduced complement deposition after monocyte depletion.

Our transcriptomic data revealed that infiltrating monocytes drive a pathogenic shift in the complement system. We sought to confirm the cellular sources and consequences of this dysregulation. Immunohistochemistry in rd10 retinas revealed that C3 protein, while expressed in aMiDMs and aMDMs, was most strongly produced by retinal Müller cells, confirmed by co-staining with Glutamine Synthetase (GS), a marker for Müller cells (Fig. 6A–B, S7A-B). This Müller cell C3 expression was significantly reduced upon monocyte ablation, indicating that infiltrating monocytes are a key upstream trigger for this glial inflammatory response (Fig. 6A–C). To confirm this at the transcriptomic level, we performed RNA-seq on the non-myeloid (CD11b-tdT-) retinal cell population, which contains Müller glia and astrocytes. In monocyte-depleted retinas, this population showed a significant downregulation of genes for *C3* and other pro-inflammatory chemokines and cytokines (*Cxcl10, Ccl12*) (Fig. 6D). Conversely, genes associated with retinal homeostasis were significantly upregulated, such as lipid homeostasis gene *Abca6*, *Etnppl*, inhibit apoptosis gene *Tmem14a*, neurotransmitter and hormones regulation gene *Glul, Mchr1, Arpp21*, and cellular homeostasis gene *Mpp3, Aredc2* (Fig. 6D). This demonstrates that preventing monocyte infiltration not only reduces inflammatory signaling from glial cells but also promotes a return to a more homeostatic state in the post-injury retina (Fig. 6E–F).

The activation of the complement cascade is tightly controlled by regulators. Our RNA-seq data from aMiDMs revealed that in the presence of infiltrating monocytes, expression of the critical alternative pathway inhibitor, Factor H (*Cfh*), was significantly decreased (Fig. 6G). We confirmed this at the protein level, showing a marked reduction of CFH protein in aMiDMs from non-depleted retinas compared to depleted controls (Fig. 6H–I). This combination of increased C3 production (from Müller cells) and decreased C3 convertase inhibition (from microglia) creates an inflammatory storm that leads to uncontrolled complement activation. Accordingly, we observed a marked increase in the deposition of the active complement fragment, C3b/iC3b, on both microglia and photoreceptors in non-depleted retinas (Fig. 6J). This pathological deposition, which tags neurons for removal, was almost completely absent in monocyte-depleted mice (Fig. 6J–K), correlating with the decreased photoreceptor degeneration observed in these animals.

## Discussion

Our study explores a central controversy in neuroimmunology by providing a definitive fate map and functional timeline for monocytes that infiltrate the CNS during neurodegeneration. Using an indelible lineage-tracing approach, we demonstrate that these cells are not merely transient inflammatory players. Instead, they exhibit remarkable plasticity, initially acting as pathogenic drivers in early stages following infiltration but later integrating into the resident myeloid population to restore homeostasis. This dual, stage-dependent role has profound implications for how we understand and potentially treat neurodegenerative diseases.

A key finding is the long-term persistence and phenotypic conversion of monocyte-derived macrophages (MDMs)^35,45,46^. We show that these cells shed their monocytic identity (CCR2, Ly6C) and acquire a full suite of morphological and transcriptomic features characteristic of microglia (TMEM119, P2RY12), a process that took place over several weeks in the mouse retina. This finding challenges the classification of CNS myeloid cells based on static marker profiling from single-cell RNA-seq, which may mistakenly group cells of different origins^25,29^. Our work establishes that what were once thought to be distinct populations can represent different developmental stages of the same lineage, shaped by the local microenvironment.

While their long-term fate is integration, their initial entry contributes to the drive toward pathogenic alterations. Our data show that these cells act as a trigger for the alternative complement pathway, a key driver of neurodegeneration^47–52^. Infiltrating monocytes orchestrate a three-part pathogenic cascade: 1) they stimulate Müller cells to produce a surge of complement C3; 2) they concurrently instruct resident microglia to downregulate the complement inhibitor CFH; and 3) this imbalance leads to the deposition of C3b/iC3b on photoreceptors, tagging them for destruction. The neuroprotective effect of ablating monocyte infiltration in both inherited and induced models of retinal degeneration validates this mechanism as a critical and targetable pathological event. Whether this robust C3 production is triggered by direct cell-to-cell contact or via paracrine signaling among infiltrating monocytes, microglia, and Müller cells remains to be fully elucidated. Our findings also shed light on the complex interactions between different myeloid populations. During the acute injury phase, resident microglia recognize and actively phagocytose a portion of the infiltrating monocytes, treating them as foreign entities. However, in chronic or resolved injury states, infiltrating cells transition to microglia-like cells that coexist with endogenous microglia. We propose that this depends on niche availability; transient injuries that cause microglial migration create an empty niche that MDMs can occupy and differentiate within, a process supported by local factors such as CSF1/IL-34^11,53,54^. In contrast, during persistent degeneration, like in the rd10 model, continuous activation prevents this stable integration, explaining the transient nature of MDMs in that context.

In conclusion, this work reveals the dynamic, dual nature of infiltrating monocytes in retinal degeneration. In the acute phase, they are potent amplifiers of complement-driven pathology^19,20,55,56^. In the chronic phase, they help enable tissue repair and immune homeostasis^18,57^. This temporal distinction is critical as it suggests that therapeutic strategies targeting monocyte infiltration, such as CCR2 antagonism^58–60^, is best timed to coincide with the acute inflammatory window to be effective without disrupting long-term repair processes. These insights provide a new framework for developing temporally tailored immunomodulatory therapies for neurodegenerative diseases in the CNS.

## Methods and materials

### Experimental Animals

CCR2-CreER knock-in mice were generated by replacing CCR2 coding sequence with those coding for CreERt2. These mice were crossed into “floxed” transgenic lines coding for the marker tdTomato (tdT, JAX lab, #007914), and diphtheria toxin subtype A (DTA, JAX Lab, #009669), and into the rd10 (JAX lab, #004297) background to generate the following lines: (1) CCR2^CreER/+^;tdT^F/+^; (2) CCR2^CreER/+^;tdT^F/+^;DTA^F/+^; (3) rd10;CCR2^CreER/+^;tdT^F/+^ (no depletion) and (4) rd10;CCR2^CreER/+^;tdT^F/+^;DTA^F/+^ (depletion). The CCR2^CreER/+^;tdT^F/+^;CX3CR1^EGFP/+^ line was generated by crossing CX3CR1-EGFP (JAX Lab, #005582) and CCR2^CreER/CreER^;tdT^F/F^ mice. CCR2-RFP (JAX lab, #017586) knock-in mice were also crossed with CX3CR1-EGFP mice to generate CCR2^RFP/+^;CX3CR1^EGFP/+^ mice. All animals were bred and housed in a National Institutes of Health animal facility under a 12-h light/dark cycle with food ad libitum. Experiments were conducted consistently with protocols (NEI606, NEI698) approved by the National Eye Institute Animal Care and Use Committee. They adhered to the ARVO Statement for the Use of Animals in Ophthalmic and Vision Research. All mice were confirmed to be free of RD8 contamination.

### Tamoxifen-Inducible Recombination and Cell Ablation

Tamoxifen (Sigma, T5648) administration was used to induce tdT and DTA expression. Tamoxifen was dissolved in corn oil for all induction protocols. Fate-mapping in adult mice: two-month-old CCR2^CreER/+^;tdT^F/+^, CCR2^CreER/+^ or CCR2^CreER/+^;tdT^F/+^;CX3CR1^EGFP/+^ mice were administered tamoxifen (400 mg/kg dose) via oral gavage (two doses, one day apart). These animals were used in subsequent experiments 1 week later. Induction/Ablation in the rd10 Model: rd10;CCR2^CreER/+^;tdT^F/+^ (no depletion) and rd10;CCR2^CreER/+^;tdT^F/+^;DTA^F/+^ (depletion) pups received daily intraperitoneal (IP) injections of tamoxifen (80–100ug/g body weight) at postnatal days 14, 15, and 16. Induction/Ablation in the NaIO_3_ Model: 400mg/kg body weight of tamoxifen was given to the CCR2^CreER/+^;tdT^F/+^ (no depletion) and CCR2^CreER/+^;tdT^F/+^;DTA^F/+^ (depletion) mice through oral gavage (two doses, one day apart).

### Analysis of Blood and Bone Marrow Granulocytes

Sample Collection and Processing: Mice were deeply anesthetized with an intraperitoneal injection of ketamine (90 mg/kg) and xylazine (8 mg/kg). Whole blood was collected via cardiac puncture from the right atrium using an EDTA-treated syringe and immediately transferred into 10 mL of ACK Lysing Buffer (Thermo Fisher Scientific) for 5–10 minutes at room temperature to lyse red blood cells. The remaining leukocytes were pelleted by centrifugation (300 × g, 5 min), washed with cold PBS, and resuspended for staining. Bone marrow was harvested by flushing the femurs and tibias with 10 mL of Hank's Balanced Salt Solution (HBSS). The resulting cell suspension was pelleted (300 × g, 10 min, 4°C), washed once with PBS, and resuspended for staining. Immunocytochemistry and Imaging: The isolated blood and bone marrow cells were incubated on ice for 20 minutes with a cocktail of primary antibodies diluted 1:30, including: anti-CD11b (eBioscience, #53-0112-82), anti-Ly6C (Thermo Fisher Scientific, #50-5931-82), and anti-CD45 (eBioscience, #30-F11). Following incubation and any necessary secondary antibody steps, cells were fixed with 2% paraformaldehyde (PFA) for 20 minutes, washed twice with PBS, and the final cell pellet was resuspended in 10 μL of PBS. The cell suspension was then smeared onto gelatincoated slides and allowed to air-dry. Slides were mounted with a DAPI-containing medium (Vector Labs) and a coverslip. Images were acquired using either an Olympus FV3000 or a Nikon Eclipse Ti2 confocal microscope.

### NaIO_3_-Induced RPE Injury

To induce acute RPE injury, two-month-old mice of the following genotypes were used: CX3CR1^GFP/+^;CCR2^RFP/+^, CX3CR1^GFP/+^;CCR2^CreER/+^;tdT^F/+^, CCR2^CreER/+^;tdT^F/+^ and CCR2^CreER/+^;tdT^F/+^;DTA^F/+^. All CreER-containing mouse lines were pre-treated with tamoxifen one week prior to injury induction. Injury was induced via a single intraperitoneal injection of sodium iodate (NaIO_3_; Honeywell Research Chemicals) at a dose of 30 mg/kg body weight. Animals were subsequently euthanized for retinal analysis at various time points ranging from 1 to 40 days post-injection.

### Light damage (LD) models

Mice were dark-adapted for 7 days prior to light exposure. Pupils were then fully dilated with a topical solution of 1% tropicamide and 2.5% phenylephrine (Alcon). Following dilation, the animals were exposed to 2 × 10^4^ lux of diffuse white fluorescent light for 2 hours, as previously described^61^. After light exposure, the mice were maintained in typical ambient cyclic light conditions (100 lux, 12:12 h), where they were housed. The mice were harvested 3 and 7 days after light damage.

### Electroretinography (ERG) detection and analysis

ERG was recorded using an Espion E3 system (Diagnosys). Mice were anesthetized with an intraperitoneal injection of ketamine (90 mg/kg) and xylazine (8 mg/kg) after dark adaptation overnight. Pupils were dilated with topically administered tropicamide (1%, Alcon) and phenylephrine (2.5%, Alcon), and proparacaine hydrochloride (0.5%, Alcon) was used for topical anesthesia. Flash ERG recordings were obtained simultaneously from both eyes with gold wire loop electrodes, with the reference electrode placed in the mouth and the ground subdermal electrode at the tail. ERG responses were obtained at increasing light intensities over the ranges of 1 × 10^− 4^ to 10 cd·s/m^2^ under dark-adapted conditions and 0.3 to 100 cd·s/m^2^ under a rod-saturating background light. The stimulus interval between flashes varied from 5 s at the lowest stimulus to 60 s at the highest stimulus. 2 to 20 responses were averaged depending on flash intensity. ERG signals were recorded with 0.3-Hz low-frequency and 300-Hz high-frequency cutoffs sampled at 1 kHz. Analysis of a-wave and b-wave amplitudes was performed using Espion ERG Data Analyzer software (version 6.0.54). The average real time wave images were generated with Prism (Version 10). The a-wave amplitude was measured from the baseline to the negative peak, and the b-wave was measured from the a-wave trough to the maximum positive peak. Statistical comparisons of ERG amplitudes between animals of different genotypes were analyzed using a two-way ANOVA.

### In Vivo Retinal Imaging with Spectral-Domain Optical Coherence Tomography (SD-OCT)

Image Acquisition: Prior to imaging, mice were anesthetized with an intraperitoneal injection of ketamine (90 mg/kg) and xylazine (8 mg/kg), and pupils were dilated with topical 1% tropicamide and 2.5% phenylephrine. Retinal structure was assessed using a Bioptigen SD-OCT imaging system (InVivoVue Software). For each eye, volume scans were acquired, centered on the optic nerve head. Each volume consisted of 4 sequential radial B-scans (each composed of 1,000 A-scans, averaged 40 times) covering a 1.4 × 1.4 mm area. Image Analysis and Quantification: Retinal layer thickness was quantified using the manufacturer's analysis software (Bioptigen; Diver). Measurements were taken in the superior and inferior quadrants at a radial distance of 0.6 mm from the optic nerve head and then averaged for each eye. Following manual segmentation of the retinal layers, the thickness of two key regions was measured: Entire Retinal Layer (ERL): Defined as the distance from the nerve fiber layer to the apical side of the RPE. Outer Retinal Layer (ORL): Defined as the distance from the outer plexiform layer (OPL), inclusive, to the apical side of the RPE. B-scans containing imaging artifacts or shallow retinal detachments were manually identified and excluded from the analysis

### Flow cytometry assay

The blood was collected from the right atrium, and the bone marrow cells were splashed from the tibia as described above (Analysis of Blood and Bone Marrow Granulocytes). The mice include no tamoxifen induction, 1 day after tamoxifen induction of both CCR2^CreER/+^;tdT^F/+^ (no depletion) and CCR2^CreER/+^;tdT^F/+^;DTA^F/+^ (depletion) mice. After digestion in ACK buffer (Gibco), the cells were stained with Ly6C-PerCP Cyanine5.5 and CD11b 488. After washing, the 500ul of suspended cells were analyzed using a Beckman Coulter CytoFlex NUV (Brea, CA) flow cytometer.

### Immunohistochemistry and Confocal Imaging

Fixed eyecups were prepared for the whole retina flat mount and cryosections. The retinal samples were treated with 1% Triton for 1 hr for the flat mount and with 0.25% Triton in PBST (0.25% Tween 20) for 0.5 hrs for the retinal section. After blocking, the following primary antibodies were used: Iba1 ( 1:500 Wako), Iba1 (SYSY, 311H9H4, 1:300), CD11b (BioRad, 1:100), Ki67 (eBioscience, # 50-5698-82, 1:40), TMEM119 (Synaptic System, 1:500), anti-P2RY12 (1:100, ThermoFisher, #PA5–77671 and Sigma, #HPA014518), anti-mouse CD68 (1:200, BioRad, #MCA1957), anti-mouse CD45 (1:100, Bio-Rad, #MCA1388), Ly6C (ThermoFisher Scientific, clone#RB6–8C5, 1:100), MHC2 (IA/IE, BD Bioscience, 1:30), cone Arestin (1:200, Millipore, #AB15282), Glutamate synthesis (GS, 1:200, Millipore), CD74 (1:100, clone number:VIC-Y1, Thermofisher), Lectin PNA From Arachis hypogaea (peanut, ThemoFisher), C3 (1:200, Hycult, HM1045), C3/iC3b/C3C (1:200, Hycult, HM1078), C3b/iC3b/C3C (1:100, Hycult, clone 2/11, HM1065). Secondary antibodies raised in goat or donkey and conjugated to either Alexa 488, Alexa 568 or Alexa 647 (1:300, ThermoFisher and Jax Lab) were used. The images were taken with the Olympus 1000/3000 and Nikon Eclipse Ti2 confocal microscope. For all quantitative mean fluorescence intensity analyses, images were acquired exclusively on the Olympus FV3000 confocal microscope. All images within a single experiment were captured using identical, fixed imaging parameters (e.g., laser power, gain, offset) to ensure comparability. Analysis was then performed using Fiji/ImageJ.

### Morphological and Fluorescence Intensity Quantification

All quantitative image analysis was performed using Fiji (ImageJ). Sholl Analysis for Morphological Quantification: To quantify microglial morphology and process ramification, a Sholl analysis was performed using the dedicated plugin in Fiji. For each cell, a high-resolution confocal image was processed by applying a threshold, converting to a binary image, and then skeletonizing the processes. The center of the cell soma was manually defined, and the analysis was configured with concentric circles at 10 μm intervals up to a final radius of 120 μm. The number of intersections between the skeletonized processes and each concentric circle was recorded. For each experimental group, a total of 16–20 cells from at least 4 different biological retinas were analyzed. The resulting data were used to calculate correlation coefficients and were plotted using GraphPad Prism (V10). Mean Fluorescence Intensity Quantification: To quantify protein expression levels, the mean fluorescence intensity was measured from immunolabeled cells. For each experimental condition, regions of interest (ROIs) were manually drawn around randomly selected cells or areas in the acquired confocal images. The Mean Gray Value within each ROI was then measured. This value, representing the average fluorescence intensity per pixel for that cell, was used to compare groups statistically.

### Isolation of Retinal Myeloid Populations by FACS

Retinal microglia and monocyte-derived macrophages (MDMs) were isolated for RNA analysis using a protocol adapted from a previous study^62^. Retinal Dissociation: Mice from the relevant experimental groups (rd10 non-depletion and depletion groups at P19/P23; NaIO_3_-injury model at day 40) were euthanized, and eyes were immediately enucleated into ice-cold Hank’s Balanced Salt Solution (HBSS). Retinas were dissected, transferred into a 0.2% papain solution, and incubated first at 8°C for 45 minutes, followed by a 7-minute incubation at 28°C to achieve dissociation. The reaction was then neutralized with a solution containing albumin, glucose, and DNase I. The resulting single-cell suspension was pelleted and washed once. Cell Staining for FACS: The cell pellet was resuspended in 100 μL of staining buffer (BD Pharmingen, #554656) containing a FITC-conjugated antibody to CD11b (1:50; eBioscience, #11–0112). The suspension was incubated on ice for 20 minutes. Following incubation, cells were washed twice with 10 mL of staining buffer supplemented with 2 mM EDTA. The final cell pellet was resuspended in 0.5 mL of staining buffer containing EDTA and DAPI for viability staining. Cell populations were sorted using a BD FACSAria II Flow Cytometer at the NHLBI Flow Cytometry Core Facility. The following gating strategy was applied: Resident Microglia/MiDMs: CD11b+ & tdTomato-; Monocyte-Derived Macrophages/MDMs: tdTomato+; Negative Population (for control): CD11b- & tdTomato-. Each sorted cell population was collected directly into a 2 mL tube containing 1 mL of TRIzol reagent (Thermo Fisher Scientific). Samples were then immediately stored at −80°C for subsequent RNA isolation.

### Bulk RNA sequencing

The total RNA was isolated from sorted cells using Trizol (Invitrogen) according to the manufacturer's instructions. Briefly, 0.2 mL of chloroform (Sigma) was added to 1 ml of Trizol with the cells and incubated for 3 minutes at room temperature. Centrifuge the samples at 3500g for 40 minutes at 4°C. Transfer 400–500 μL of the aqueous phase to a fresh tube and add an equal volume of 70% ice-cold ethanol. Then, following the RNA extraction kit (Qiagen, Qiagen RNeasy Plus Micro kit) for binding RNA and purification. RNA quality and quantity were measured using a Bioanalyzer (Agilent 2100). RNAs with RIN values > 7 were used for RNA-seq. The RNA-seq was performed at AmpSeq Biotechnology company (Gaithersburg, MD, USA). The cDNA library was prepared using the VAHTS Universal V10 RNA-seq Library Prep Kit for Illumina (Vazyme). The quality was assessed using Qubit (ThermoFisher) and qPCR, and the fragment size was analyzed with Qsep. The sequencing was run on Element Biosciences AVITI (Element Biosciences) using a 2×150 cycle sequencing kit (Illumina). The RNASeq quantification pipeline begins by using fastp (fastp, v0.23.1) to trim adapters and low-quality bases from the raw sequencing reads. Next, the trimmed reads are aligned to the mouse reference genome (mm39.fa) using the STAR (v2.7.11) aligner, which efficiently maps the reads to their corresponding genomic locations. Following alignment, the Salmon package (v1.10.2) is employed to perform quantification at the transcript level. Salmon employs a lightweight alignment-based approach to estimate transcript abundances, considering both the mapped reads and the known transcript sequences. Finally, the tximport package (v1.30.0) in R is utilized to import the transcript-level quantification results generated by Salmon and summarize them at the gene level. This process involves mapping the transcripts to their corresponding genes and aggregating the abundance estimates, resulting in gene-level quantification data that can be used for downstream analysis and interpretation.

Following the trimming step, quality control is performed on the trimmed sequences using FastQC (v0.11.9). To summarize the FastQC results across all samples, MultiQC (v1.11) is employed. MultiQC aggregates the individual FastQC reports and generates a single, interactive report that allows for easy comparison and visualization of the QC metrics across the entire dataset. The gene-level quantification data includes two main types of quantification measures: raw count data and TPM (Transcripts Per Million) data. Raw count data represents the number of reads that map to each gene, providing a direct measure of gene expression. TPM data, on the other hand, is a normalized measure of gene expression that takes into account the length of each gene and the total number of mapped reads in each sample. TPM values are calculated by dividing the read counts by the length of each gene in kilobases, and then normalizing the resulting values to account for differences in library size across samples. TPM data allows for more accurate comparison of gene expression levels between samples and across different genes. Both raw count data and TPM data can be used for downstream analysis, such as differential expression analysis (DESeq2).

### Gene Set Enrichment Analysis (GSEA) and single sample GSEA (ssGSEA)

GSEA version 4.3.3 was used. The gene sets database used the mouse collection (MSig DB), select mh.all.v2026.1.Mm.symbols.gmt. The number of permutations is 1000. Chip platform is Mouse_Gene_Symbol_Remapping_MSigDB. For ssGSEA, version 10.1.0 was used. The gene sets database files used m5.go.bp.v2026.1.Mm.Symboles.gmt. The weighting exponent is 0.75, and the minimum gene set size is 10.

### Statistical Analysis

All statistical analyses were conducted using GraphPad Prism (Version 10). Data are presented as mean ± SEM (standard error of the mean), and all experiments were performed with at least three biological replicates unless otherwise noted. For comparisons between two experimental groups, an unpaired, two-tailed Student’s t-test was used. In cases where data did not follow a Gaussian distribution, a non-parametric Mann-Whitney test was applied instead. Morphological comparisons derived from Sholl analysis were assessed using a correlation coefficient. All electroretinography (ERG) data involving multiple stimuli were analyzed using a two-way ANOVA, followed by an appropriate post hoc test for multiple comparisons. A p-value of less than 0.05 was considered statistically significant. Significance is denoted in figures as *p < 0.05, **p < 0.01, and ***p < 0.001.

## Supplementary Material

Supplementary Files

This is a list of supplementary files associated with this preprint. Click to download.

- Supplementfile1.microgliaenrichedgenesforclusteranalysis.xlsx
- Supplementfile2.GeneexpressionMicrogliavsMDM.xlsx
- Supplementfile3.GOssGEEAMDMMicrogliaMiDM.xlsx
- Supplementfile4.GSEAaMiDMnodepletionvsdepletion.xlsx
- Supplementfile5.GSEAaMDMvsaMiDm.xlsx
- Graphabstract.jpg
- Fig.S1.jpg
- Fig.S2.jpg
- Fig.S3.jpg
- Fig.S4.jpg
- Fig.S5.jpg
- Fig.S6.jpg

The supplementary materials contain S1 to S7 figures and figure legends. Other Supplementary Material for this manuscript includes supplement files 1–5.

## Data Availability

The datasets generated and/or analyzed in the current study are available in the SRA at NCBI under project ID PRJNA1368926. The data will be accessible on December 1, 2026.

## Highlights

- **Homeostasis:** In the healthy retina, resident microglia maintain a surveillance state, characterized by ramified morphology and an evenly tiled distribution.
- **Acute Pathology:** During acute injury, infiltrating monocytes trigger a pathogenic cascade: they induce Müller glia to produce complement C3 while reprogramming microglia to downregulate the inhibitor CFH, leading to unchecked complement activation and photoreceptor death.
- **Chronic Resolution:** In the chronic stable phase, surviving monocyte-derived macrophages (MDMs) integrate with resident microglia-derived macrophages (MiDMs), acquiring a microglia-like phenotype to establish a new, stable myeloid homeostasis.
